# Supplementary material for: The Discovery of Potential Repellent Compounds for Zeugodacus cucuribitae (Coquillett) from Six Non-Favored Hosts
Source: Int J Mol Sci. 2025 Jul 8;26(14):6556. doi: 10.3390/ijms26146556 (PMC12295180; doi:10.3390/ijms26146556)
Supplement: Supplementary file 1 [file ijms-26-06556-s001.zip › Figure s1 Construction of phylogenetic tree based on gene sequences of Z. cucuribitae and several Diptera insects.pdf]

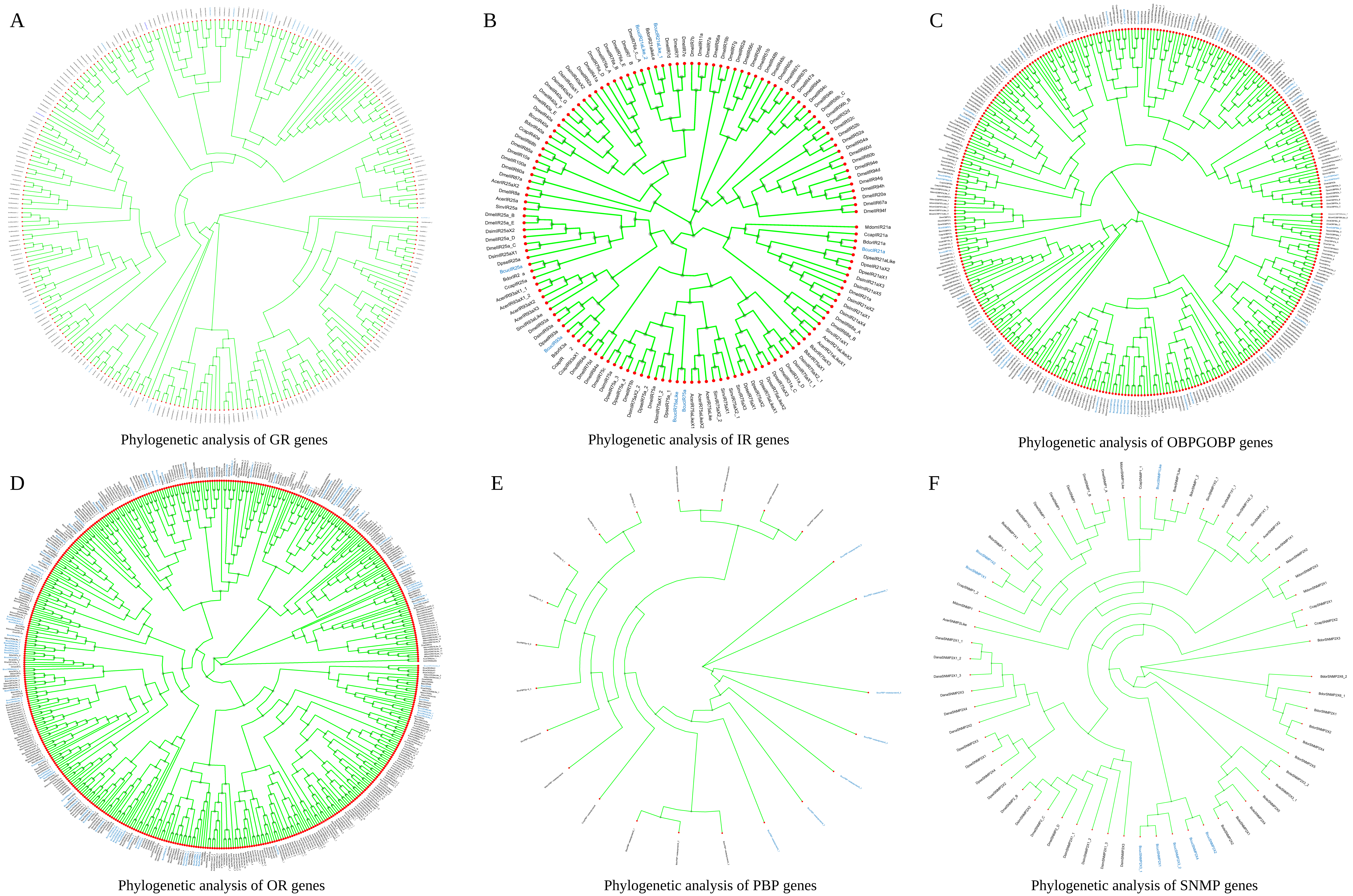
A
Phylogenetic analysis of GR genes
B
Phylogenetic analysis of IR genes
C
Phylogenetic analysis of OBPGOBP genes
D
Phylogenetic analysis of OR genes
E
Phylogenetic analysis of PBP genes
F
Phylogenetic analysis of SNMP genes
